# Investigation of the Thermal Conductance of MEMS Contact Switches

**DOI:** 10.3390/mi16080872

**Published:** 2025-07-28

**Authors:** Zhiqiang Chen, Zhongbin Xie

**Affiliations:** School of Mechano-Electronic Engineering, Xidian University, Xi’an 710071, China; xiezhongbin@stu.xidian.edu.cn

**Keywords:** microelectromechanical systems (MEMS), surface roughness, thermal conductance, interface interaction

## Abstract

Microelectromechanical system (MEMS) devices are specialized electronic devices that integrate the benefits of both mechanical and electrical structures. However, the contact behavior between the interfaces of these structures can significantly impact the performance of MEMS devices, particularly when the surface roughness approaches the characteristic size of the devices. In such cases, the contact between the interfaces is not a perfect face-to-face interaction but occurs through point-to-point contact. As a result, the contact area changes with varying contact pressures and surface roughness, influencing the thermal and electrical performance. By integrating the CMY model with finite element simulations, we systematically explored the thermal conductance regulation mechanism of MEMS contact switches. We analyzed the effects of the contact pressure, micro-hardness, surface roughness, and other parameters on thermal conductance, providing essential theoretical support for enhancing reliability and optimizing thermal management in MEMS contact switches. We examined the thermal contact, gap, and joint conductance of an MEMS switch under different contact pressures, micro-hardness values, and surface roughness levels using the CMY model. Our findings show that both the thermal contact and gap conductance increase with higher contact pressure. For a fixed contact pressure, the thermal contact conductance decreases with rising micro-hardness and root mean square (RMS) surface roughness but increases with a higher mean asperity slope. Notably, the thermal gap conductance is considerably lower than the thermal contact conductance.

## 1. Introduction

Contact phenomena are crucial in a wide range of engineering applications, especially in microelectromechanical system (MEMS) devices. At its core, contact refers to the interaction between two surfaces coming together, forming a junction where mechanical, thermal, and electrical properties are influenced by the surface characteristics of the materials involved. The nature of this contact can significantly impact the performance and reliability of MEMS devices, which are increasingly used in applications spanning telecommunications, automotive systems, and beyond [1,2,3,4,5,6,7,8,9]. In the case of MEMS switches, contact is particularly important for ensuring reliable and efficient operation. These switches mainly rely on electrostatic actuation to open and close circuits, and the quality of the contact between the switch elements directly affects key performance metrics such as insertion loss, isolation, and overall functionality [10,11]. High-quality contacts ensure minimal resistance, which facilitates efficient signal transmission and reduces power consumption [12]. In contrast, poor contact can lead to increased thermal and electrical resistance, resulting in degraded performance and, in some cases, device failure [13,14]. Therefore, understanding and optimizing contact behavior is essential for advancing MEMS technology.

In recent years, research into thermal contact resistance (TCR) has gained significant attention, as it plays a pivotal role in heat transfer within MEMS devices [15,16,17]. TCR arises due to the microscopic roughness of contacting surfaces, which reduces the actual contact area compared to the apparent contact area [18,19]. This phenomenon is especially relevant in MEMS, where surface roughness and the scale of contact can have a dramatic effect on thermal management [20]. As devices shrink in size, the influence of TCR becomes more pronounced, making it critical to understand the mechanisms at play [21,22,23,24]. Models for thermal contact conductance between rough surfaces are typically classified into three types: elastic [25], plastic [18], and elasto-plastic [26]. Studies have shown that during the operation of MEMS switches, material transfer occurs at the contact interface with repeated actuation, leading to changes in surface roughness [27,28,29]. In other words, each contact event results in permanent deformation. Therefore, modeling the contact in MEMS switches as purely elastic is not appropriate, as the surface morphology evolves over time with continued use. As a result, a plastic contact model is adopted in this study to investigate the thermal transport across the contact interface of MEMS switches. One of the foundational plastic models in the study of TCR is the Cooper–Mikic–Yovanovich (CMY) model, which offers a framework for predicting thermal contact conductance between rough surfaces. The CMY model assumes that the contact surfaces are conforming and rough, with roughness characterized by a Gaussian distribution [18,30,31,32]. This model has become widely used due to its ability to correlate experimental data with theoretical predictions, establishing it as a cornerstone in the study of thermal contact resistance [26,33,34]. According to the CMY model, thermal contact conductance depends on several factors, including the apparent contact pressure, the effective surface roughness, and the micro-hardness of the materials involved [18]. The model expresses thermal contact conductance as a function of the relative mean plane separation and the effective contact pressure, enabling the prediction of TCR under various loading conditions [19]. Over the past half-century, the CMY model has been refined to accurately describe heat transfer behavior between non-smooth contact surfaces. As a result, it has become widely used in numerous fields, serving as a key tool in advancing the understanding of thermal contact resistance in MEMS devices [19,34,35,36,37,38].

Despite its widespread use, the CMY model has its limitations. One major issue is its reliance on the assumption of plastic deformation at the contact interface, which may not accurately capture the behavior of all materials under varying load conditions. For example, in the case of hard materials, the model may overestimate the actual contact area, leading to an inflated prediction of thermal conductance [39,40]. However, as mentioned above, repeated actuation leads to permanent surface changes and material transfer, making purely elastic or even elasto-plastic models less suitable. The assumption of plastic contact, central to the CMY model, aligns well with these observed phenomena. Additionally, the model does not account for the effects of surface contaminants, oxide layers, or other interfacial phenomena that can significantly impact thermal contact resistance [39]. Another limitation of the CMY model is that it has primarily been validated at room temperature, leaving a gap in our understanding of its applicability at low temperatures, such as those encountered in cryogenic environments [41]. Recent studies have shown that the thermal properties of materials can change considerably at low temperatures, potentially affecting the accuracy of the CMY model in predicting TCR. For instance, research by Maddren and Marschall highlighted discrepancies between the predictions of the CMY model and experimental results at cryogenic temperatures, suggesting that the model may need adjustments to better account for the unique behaviors of materials in these conditions [42]. Our current study focuses on contact behavior under conditions close to ambient temperature, where this assumption remains reasonably valid in this paper. Additionally, there are currently some applications of the CMY model in the micro/nano field [43,44], which further validates its feasibility in describing the thermal conductivity of MEMS structures.

In this paper, we systematically investigate the regulation mechanism of thermal conductance in MEMS contact switches by combining the CMY model with finite element simulations. We examine how contact pressure, micro-hardness, root mean square (RMS) surface roughness, and other key parameters influence thermal conductance. The findings provide crucial theoretical support for enhancing the reliability and optimizing the thermal management of MEMS contact switches.

## 2. Theoretical Model

**Figure 1:**
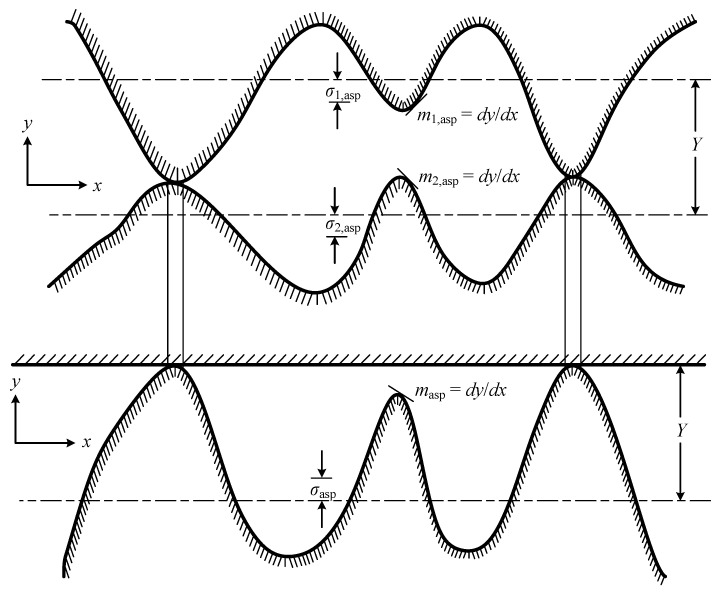
Typical contact joint between two conforming rough surface. $\sigma_{1,\mathrm{asp}}$ and $\sigma_{2,\mathrm{asp}}$ are the RMS surface roughness and $m_{1,\mathrm{asp}}$ and $m_{2,\mathrm{asp}}$ are the mean absolute asperity slope of two contacting surfaces, respectively. $\sigma_{\mathrm{asp}}$ and $m_{\mathrm{asp}}$ are the effective RMS surface roughness of asperities and the effective absolute mean asperity slope for a typical joint formed by two conforming roughness surfaces.

Figure 1 is the conforming rough surface model developed by Cooper, Mikic and Yovanovich in 1969 [18]. In this model, they assumed that the surface asperities have a Gaussian height distribution around a mean contact plane, and that the asperities are randomly distributed across the apparent contact area. $\sigma_{1,\mathrm{asp}}$ and $\sigma_{2,\mathrm{asp}}$ are defined in references [18,34] in detail, and $\sigma_{\mathrm{asp}}$ and $m_{\mathrm{asp}}$ can be expressed as:

(1) $$\sigma_{\mathrm{asp}}=\sqrt{\sigma_{1,\mathrm{asp}}^{2}+\sigma_{2,\mathrm{asp}}^{2}}$$

(2) $$m_{\mathrm{asp}}=\sqrt{m_{1,\mathrm{asp}}^{2}+m_{2,\mathrm{asp}}^{2}}$$

Therefore, according to the CMY model, the plastic thermal contact or constriction conductance, $h_{c}$, can be expressed as [33]:

(3) $$h_{c}=1.25k_{s}\frac{m_{\mathrm{asp}}}{\sigma_{\mathrm{asp}}}{\left(\frac{P}{H_{c}}\right)}^{0.95}$$

where *P* is the contact pressure, $H_{c}$ is the micro-hardness of the softer materials, $k_{s}$ is the harmonic mean thermal conductivity of the joint, which can be expressed as:

(4) $$k_{s}=\frac{2k_{1}k_{2}}{k_{1}+k_{2}}$$

in which $k_{1}$ and $k_{2}$ represent the thermal conductivities of the surfaces. Equation (3) is applicable in the range: $10^{-6}\leq P/H_{c}\leq 2.2\times 10^{-2}$ [34].

Furthermore, the thermal gap conductance, $h_{g}$, due to the interstitial gas between the two joints is defined as [43,45]:

(5) $$h_{g}=\frac{k_{\mathrm{gas}}}{Y_{\mathrm{gap}}+M_{\mathrm{gas}}}$$

where $k_{\mathrm{gas}}$ is the thermal conductivity of the gas, and $Y_{\mathrm{gap}}$ is the distance between the mean planes, which can be expressed by [46]:

(6) $$Y_{\mathrm{gap}}=\sqrt{2}\sigma_{\mathrm{asp}}erfc^{-1}\left(\frac{2P}{H_{c}}\right)$$

where $erfc^{-1}\left(x\right)$ is the inverse function of the complementary error function. $M_{\mathrm{gas}}$ is a gas parameter used to account for rarefied gas effects [43,45]:

(7) $$M_{\mathrm{gas}}=\alpha \beta \Lambda$$

in which α is an accommodation parameter (approximately equal to 1.7 for air and clean metals), β is a fluid property parameter (equal to approximately 1.7 for air and other diatomic gases), and Λ is the mean free path of the gas (equal to approximately 0.06 μm for air at atmospheric pressure and 15 °C), which can expressed as [43,45]:

(8) $$\Lambda =\frac{k_{B}T_{g}}{2\sqrt{2}\pi D^{2}p_{g}}$$

where $k_{B}$ is the Boltzmann constant, $T_{g}$ is the gas temperature, *D* is the average diameter of the gas particles, and $p_{g}$ is the gas pressure.

In addition to thermal contact conductance and thermal gap conductance, thermal radiative conductance also exists due to thermal radiation. However, both existing studies [34,47] and simulation results consistently show that thermal radiative conductance is significantly smaller than contact and gap conductance. As a result, its impact on the overall thermal joint conductance can be considered negligible and is neglected during the following analysis.

Therefore, the thermal joint or total conductance is obtained by adding Equations (3) and (5) if the thermal radiative conductance is ignored. The thermal joint conductance ($h_{j}$) is expressed as:

(9) $$h_{j}=h_{c}+h_{g}=1.25k_{s}\frac{m_{\mathrm{asp}}}{\sigma_{\mathrm{asp}}}{\left(\frac{P}{H_{c}}\right)}^{0.95}+\frac{k_{\mathrm{gas}}}{Y_{\mathrm{gap}}+M_{\mathrm{gas}}}$$

Then, the thermal joint resistance becomes:

(10) $$R_{j}=\frac{1}{h_{j}A_{a}}={\left\{\left[1.25k_{s}\frac{m_{\mathrm{asp}}}{\sigma_{\mathrm{asp}}}{\left(\frac{P}{H_{c}}\right)}^{0.95}+\frac{k_{\mathrm{gas}}}{Y_{\mathrm{gap}}+M_{\mathrm{gas}}}\right]A_{a}\right\}}^{-1}$$

where $A_{a}$ is the apparent contact area.

The above equations will be used to calculate the thermal contact, the gap, and the joint conductance of two conforming surfaces in the following contents. The heat generated by the Joule effect of the current is transferred through the aforementioned thermal conductance pathways.

## 3. Finite Element Simulation

**Figure 2:**
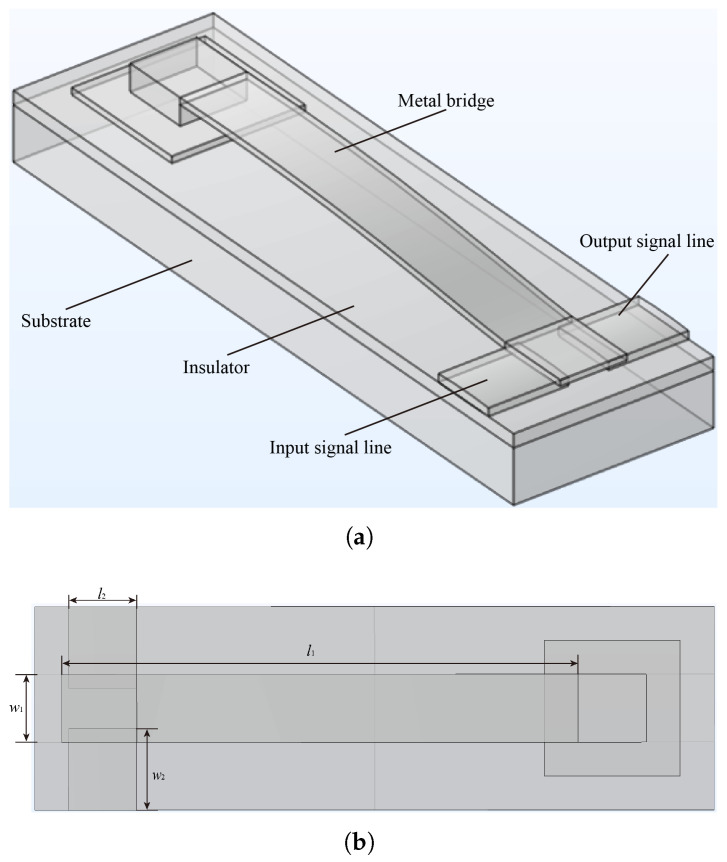
(**a**) Structure and (**b**) top view of MEMS contact switches, where $l_{1}$ and $w_{1}$ are the length and width of of the metal bridge, and $l_{2}$ and $w_{2}$ are the length and width of the input and output signal line, respectively.

Figure 2 presents the finite element method (FEM) simulation model in this study, based on reference [48]. As shown in Figure 2a, the MEMS contact switches consist of a silicon substrate with a silicon dioxide insulating layer, a metal bridge, and input and output signal lines. When the switches are in the “up” state, the input and output terminals are in an open circuit condition. In the “down” state, the signal can normally be transmitted from the input port to the output port. Since we are primarily concerned with thermal joint conductance, this paper focuses on analyzing the down-state behavior. In Figure 2b, $l_{1}$ = 86 μm, $w_{1}$ = 10 μm, $l_{2}$ = 10 μm, $w_{2}$ = 12 μm, and the thickness of the metal bridge and signal line are 1.2 and 1.0 μm, respectively.

The contact pressure of the MEMS contact switches ranges from 50 to 2500 μN according to references [12,49,50]. Given that the contact area of the switches is 3 μm × 12 μm = 36 μm^2^, the contact pressure varies from 1.38 to 69 MPa. For this study, we analyze contact pressures ranging from 1 to 10 MPa. The micro-hardness, *H_c_*, of Au is 660 MPa, as stated in reference [51]. The RMS surface roughness of gold has been reported as 10 nm in reference [3], 1.68 nm, 2.49 nm, 4.75 nm, 7.05 nm, and 9.92 nm in reference [52], and 10.315 nm in reference [53]. Accordingly, the asperity average height, $\sigma_{\mathrm{asp}}$, for gold was set between 1 and 20 nm. The asperity average slope, $m_{\mathrm{asp}}$, was set to range from 0.5 to 2.5, based on reference [43]. The substrate was fixed at its bottom surface. Contact pressure was applied to the metal cantilever in the region overlapping the metal signal line. A voltage of 0.3 mV was applied to one end of the signal line’s cross-section, while the other end was grounded, to study Joule heating and its distribution across the MEMS contact switch. The mesh had an average element quality of 0.82. The solution used a relative tolerance of 0.001, and the PARDISO solver was employed for the simulation.

## 4. Results and Discussion

### 4.1. Switch Temperature

**Figure 3:**
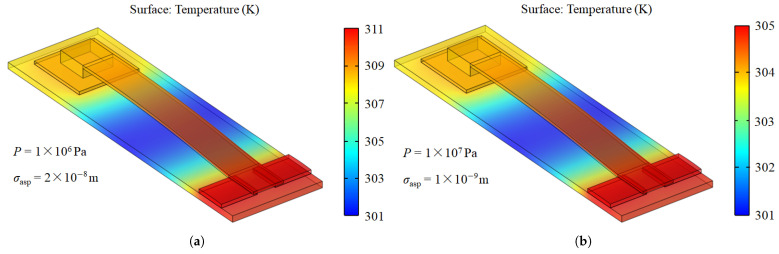
Temperature of the contact switch under different contact pressure and RMS surface roughness: (**a**) *P* = 1 MPa and $\sigma_{\mathrm{asp}}$ = 20 nm; (**b**) *P* = 10 MPa and $\sigma_{\mathrm{asp}}$ = 1 nm.

Figure 3 illustrates the simulated temperature distribution of the contact switch under varying contact pressures and RMS surface roughness values. A temperature difference of 5 K is observed between the two different simulation conditions. Since the signal line is the primary current path, the maximum temperature is observed on the signal line. Given that thermal conductance is the key factor influencing thermal conduction, we investigated how various parameters affect thermal conductance in the following contents.

### 4.2. Thermal Conductance

#### 4.2.1. Thermal Contact Conductance

**Figure 4:**
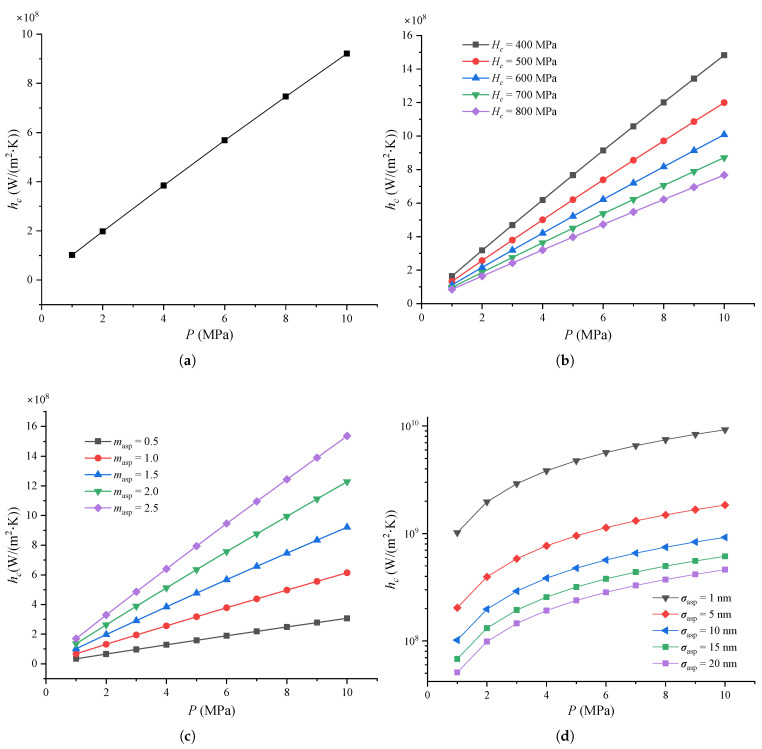
Thermal contact conductance of MEMS contact switches under different: (**a**) Contact pressure, where $H_{c}$ = 0.66 GPa, $\sigma_{\mathrm{asp}}$ = 10 nm, and $m_{\mathrm{asp}}$ = 1.5; (**b**) Micro-hardness, where $\sigma_{\mathrm{asp}}$ = 10 nm, and $m_{\mathrm{asp}}$ = 1.5; (**c**) Effective absolute mean asperity slope, where $H_{c}$ = 0.66 GPa and $\sigma_{\mathrm{asp}}$ = 10 nm; (**d**) RMS surface roughness of asperities for a typical joint, where $H_{c}$ = 0.66 GPa and $m_{\mathrm{asp}}$ = 1.5.

Figure 4 shows the thermal contact conductance of the MEMS contact switches under varying pressure and other parameters.

As shown in Figure 4a, the thermal contact conductance $h_{c}$ increases linearly as pressure *P* increases up to 10 MPa. This behavior aligns with Equation (3), where *P* is proportional to $h_{c}$. Other researchers also observed in their experiments that the thermal contact conductance of stainless steel increases with rising contact pressure [54,55]. This phenomenon occurs because the contact surface is not perfectly smooth, and as the contact pressure increases, more peaks on the metal beam make contact with the signal line, thereby increasing the contact area. Therefore, the thermal contact conductances increases as the contact pressure increases.

Figure 4b shows the thermal contact conductance under varying micro-hardness $H_{c}$ and pressure. It can be seen that $h_{c}$ increases linearly with *P* for different values of $H_{c}$ as *P* increases from 1 to 10 MPa. Notably, a lower micro-hardness leads to a faster increase in thermal contact conductance. Additionally, for the same pressure, lower micro-hardness results in higher thermal contact conductance. This is because softer materials have more asperities on the metal surface, which come into contact with the signal line and deform plastically under the same pressure, increasing the contact area.

Figure 4c shows the thermal contact conductance for different pressures and effective absolute mean asperity slopes. As with Figure 4b, $h_{c}$ increases linearly as pressure increases from 1 to 10 MPa, and larger mean asperity slopes lead to higher thermal contact conductance values at the same pressure. As defined in references [18,34], a larger mean asperity slope corresponds to steeper asperities between the contact surfaces.

Finally, Figure 4d shows the thermal contact conductance at different pressure and RMS surface roughness values. It is evident that $h_{c}$ increases with *P* at the same $\sigma_{\mathrm{asp}}$. In fact, $h_{c}$ increases linearly with *P* for all values of $\sigma_{\mathrm{asp}}$ along the linear axis. Other researchers have also found that thermal conductance decreases as RMS surface roughness increases, given the same contact pressure [54]. Similar findings were obtained by Kumar et al. [55] that the thermal contact conductance decreased as the surface roughness increased for the given temperature at Al-Al contact surfaces. This is mainly because higher surface roughness results in a smaller contact area at the same pressure and asperity slope, leading to a reduction in the thermal contact conductance.

#### 4.2.2. Thermal Gap Conductance

**Figure 5:**
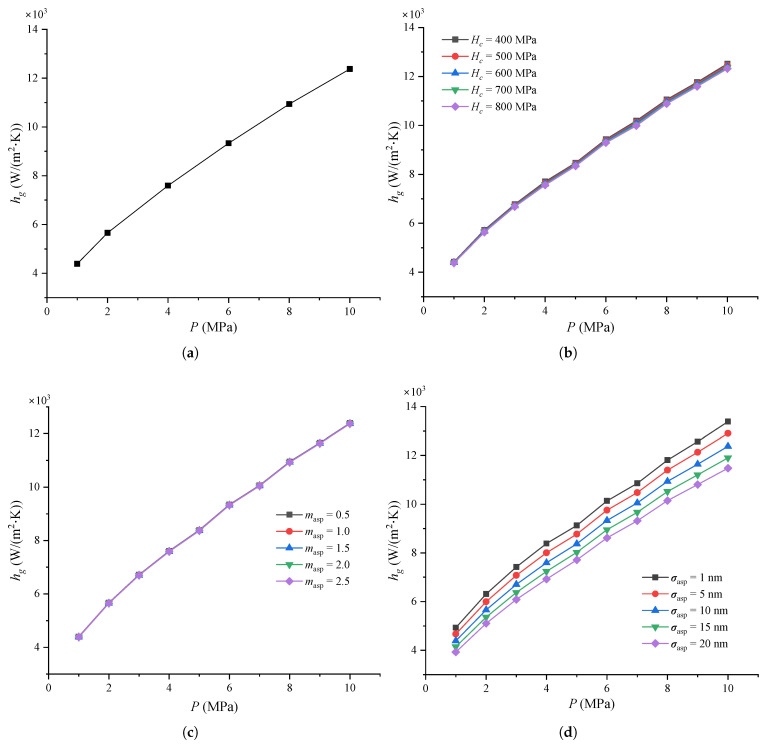
Thermal gap conductance of MEMS contact switches under different: (**a**) Contact pressure, where $H_{c}$ = 0.66 GPa, $\sigma_{\mathrm{asp}}$ = 10 nm, and $m_{\mathrm{asp}}$ = 1.5; (**b**) Micro-hardness values, where $\sigma_{\mathrm{asp}}$ = 10 nm, and $m_{\mathrm{asp}}$ = 1.5; (**c**) Effective absolute mean asperity slope values, where $H_{c}$ = 0.66 GPa and $\sigma_{\mathrm{asp}}$ = 10 nm; (**d**) RMS surface roughness values of asperities for a typical joint, where $H_{c}$ = 0.66 GPa and $m_{\mathrm{asp}}$ = 1.5.

Figure 5 shows the thermal gap conductance of MEMS switches under different parameters.

From Figure 5a, we can observe that $h_{g}$ almost linearly increases from approximately 4.5 × 10^3^ to 12.5 × 10^3^ W/($m^{2}\cdot$K) as *P* increases from 1 to 10 MPa. The variation is explained by Equations (5)–(8). As shown in Equations (7) and (8), the gas parameter remains constant for a specified gas; therefore, it does not change as the contact pressure. The gas used in this simulation is air at 1 atm ($1.01325\times 10^{5}$ Pa) and 15 °C, then $M_{\mathrm{gas}}$ = 173.4 nm. Therefore, the variation in the thermal gap conductance is due to the distance between the mean planes. It is important to note that the maximum thermal gap conductance of 17 × 10^3^ W/($m^{2}\cdot$K) is much smaller than the thermal contact conductance, which typically ranges from 10^8^ to 10^9^ W/($m^{2}\cdot$K). The reason for this is that the thermal gap conductance is primarily governed by heat conduction through the air, while the thermal contact conductance is dominated by heat conduction through the gold material. Yovanovich [34] also found that the thermal gap conductance of helium gas increased from about $10^{3}$ W/($m^{2}\cdot$K) to more than $10^{4}$ W/($m^{2}\cdot$K) when the applied pressure increased from 10 to 1000 torr for conforming rough Ni 200 surfaces. Other researchers also found that the thermal contact conductance increases with increase in the contact pressure [56,57,58].

**Figure 6:**
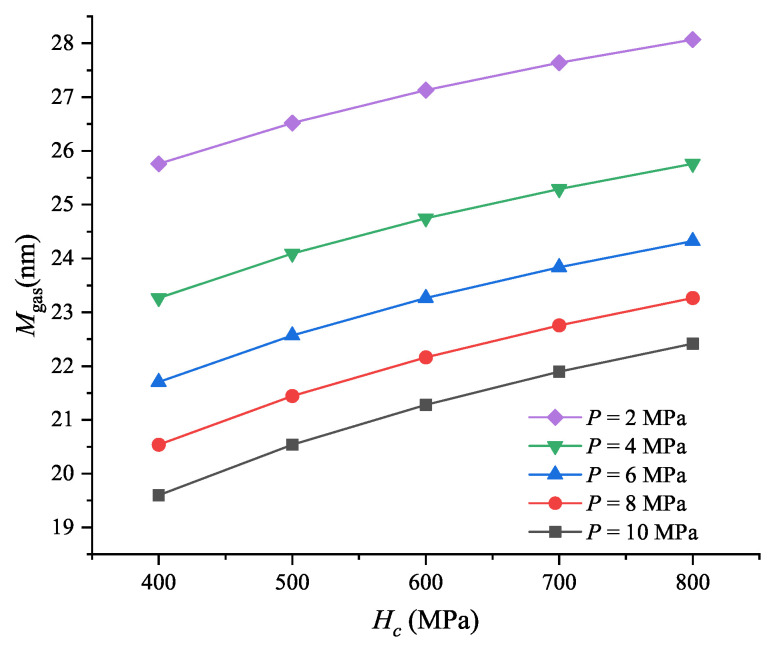
Gas parameter under different micro-hardness and pressures, where $\sigma_{\mathrm{asp}}$ = 10 nm.

Figure 5b shows the thermal gap conductance of MEMS contact switches under different micro-hardness. It can be seen that $h_{g}$ has almost no variation as $H_{c}$ for the given *P*. Figure 6 shows the reason for this phenomenon. It can be seen that $M_{\mathrm{gas}}$ increases less than 3 nm when $H_{c}$ increases from 400 to 800 MPa for the given contact pressure, while $M_{\mathrm{gas}}$ equals to 173.4 nm, which is much larger than the increment in $M_{\mathrm{gas}}$. Therefore, the thermal gap conductance remains almost constant at different micro-hardness for the given contact pressures.

Figure 5c shows the thermal gap conductance of MEMS contact switches under different mean asperity slope values. It can be seen that $h_{g}$ has no relationship with $M_{\mathrm{asp}}$. Equation (6) also shows this result.

Figure 5d presents the thermal gap conductance under varying contact pressure and RMS surface roughness. We observe that $H_{g}$ decreases slightly with increasing RMS surface roughness for given *P*. This indicates that RMS roughness has a minimal impact on the thermal gap conductance of MEMS contact switches. Equation (6) gives the reason for this phenomenon. It can be seen that $Y_{\mathrm{gap}}$ is linearly related to $\sigma_{\mathrm{asp}}$ for given pressure and micro-hardness. Similar results have been reported by other researchers [19].

It can be seen from Figure 5 that the thermal gap conductance is much smaller than the thermal contact conductance. Typically, the thermal gap conductance can be neglected when the contact pressure is relatively high, and/or the interstitial gaseous medium is a relatively poor thermal conductor. However, heat conduction across gaps becomes particularly significant when the contact pressure is relatively low or when the interstitial gas has high thermal conductivity [59]. Studies have shown that when the contact pressure is below 1 MPa, the heat transfer through solid contact points is minimal, with the majority of heat being conducted through the gas [60]. This remains true even for soft and highly conductive materials like aluminum. In many practical scenarios, particularly in electronic components, contact pressures tend to be in the low to moderate range. For example, in a bolted thermal contact between a base plate and a heat sink in a multiple chip unit (MCU) of a computer, the average contact pressure is around 0.9 MPa. Under such conditions, heat conduction through the gaseous gap becomes more influential than conduction through solid contact points [58].

#### 4.2.3. Thermal Joint Conductance

**Figure 7:**
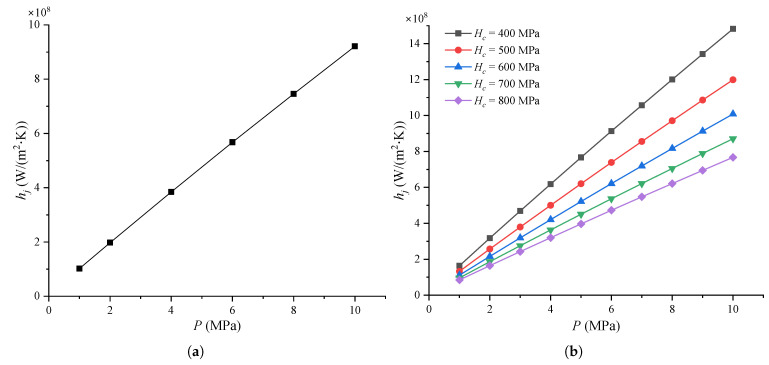

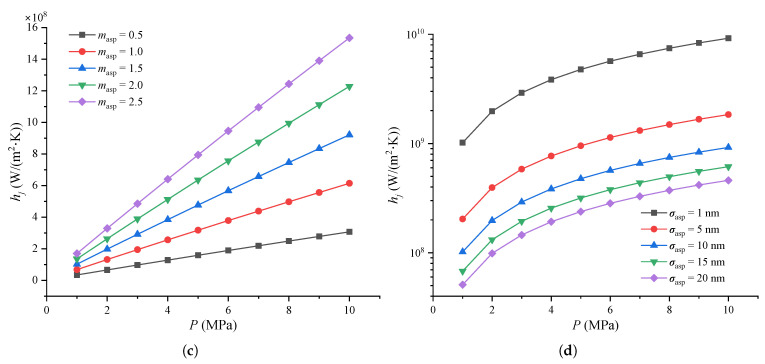
Thermal joint conductance of MEMS contact switches under different: (**a**) Contact pressure, where $H_{c}$ = 0.66 GPa, $\sigma_{\mathrm{asp}}$ = 10 nm, and $m_{\mathrm{asp}}$ = 1.5; (**b**) Micro-hardness, where $\sigma_{\mathrm{asp}}$ = 10 nm, and $m_{\mathrm{asp}}$ = 1.5; (**c**) Effective absolute mean asperity slope, where $H_{c}$ = 0.66 GPa and $\sigma_{\mathrm{asp}}$ = 10 nm; (**d**) RMS surface roughness of asperities for a typical joint, where $H_{c}$ = 0.66 GPa and $m_{\mathrm{asp}}$ = 1.5.

Figure 7 shows the thermal joint conductance of the MEMS contact switches under different parameters. It can be observed that Figure 4 and Figure 7 are nearly identical, which can be explained by Equation (9). The thermal joint conductance is composed of both the thermal gap conductance and the thermal contact conductance. However, the thermal gap conductance is significant smaller than the thermal contact conductance, meaning that the gap conductance contributes only minimally to the overall thermal joint conductance. In practical engineering applications, we often neglect the thermal gap conductance and assume that the thermal joint is equivalent to the thermal contact conductance. This assumption holds, as considering the bulk properties of the layer yields similar results [61]. Experimental results have shown that the thermal conductance at Al/MgO thin film interfaces increases from approximately 2 × $10^{8}$ to 1 × $10^{9}$ W/($m^{2}\cdot$K) as contact pressure increases [62]. Additionally, it was observed that the thermal conductance between an 80 nm pure aluminum film and a 500 μm silicon substrate decreases from around 2 × $10^{8}$ to 1.65 × $10^{8}$ W/($m^{2}\cdot$K) as the RMS surface roughness increases from about 1 nm to 10 nm [63].

In addition to the contact pressure, micro-hardness, effective absolute mean asperity slope, and RMS surface roughness, other factors such as temperature [19,64] and gas rarefaction [65] also influence the thermal joint conductance.

## 5. Conclusions

In this paper, we investigated the thermal conductance of MEMS contact switches under varying contact pressures, micro-hardness values, mean asperity slopes, and RMS surface roughness. Our results showed that thermal contact conductance increases linearly with contact pressure. Additionally, it decreases as the micro-hardness and RMS surface roughness increase, with the discrepancy being more pronounced at higher contact pressures. On the other hand, the thermal contact conductance increases with the mean asperity slope. Specifically, we observed that the thermal contact conductance increased by approximately 10 times when the contact pressure was raised from 1 to 10 MPa. Simultaneously, for different values of micro-hardness, mean asperity slope, and RMS roughness at the same contact pressure, the thermal contact conductance increased by roughly 7–10 times. Similar trends were found for the thermal gap conductance, though it was much smaller than the thermal contact conductance. The thermal joint conductance was primarily determined by the thermal contact conductance. Moving forward, we will continue this research by exploring the electrical and mechanical behaviors at the interfaces. At the same time, extending the model to incorporate temperature-dependent plasticity or performing experimental validation at varying temperatures would be valuable directions for future work. 

## Data Availability

Data supporting the findings of this study are available from the corresponding author upon reasonable request.
